# Exploring Student Food Behaviour in Relation to Food Retail over the Time of Implementing Ontario’s School Food and Beverage Policy

**DOI:** 10.3390/ijerph16142563

**Published:** 2019-07-18

**Authors:** Rhona M. Hanning, Henry Luan, Taryn A. Orava, Renata F. Valaitis, James K. H. Jung, Rashid Ahmed

**Affiliations:** 1 School of Public Health and Health Systems, Faculty of Applied Health Sciences, University of Waterloo, Waterloo, ON N0B 2J0, Canada; 2 Department of Geography, University of Oregon, Eugene, OR 97403, USA; 3 College of Nursing, Rady Faculty of Health Sciences, University of Manitoba, Winnipeg, MB R3T 2N2, Canada

**Keywords:** school policy, food, child and adolescent, retail density, energy, sodium, sugar, urban environment, overweight and obesity, GIS

## Abstract

Background: Canadian provincial policies, like Ontario’s School Food and Beverage Policy (P/PM 150), increasingly mandate standards for food and beverages offered for sale at school. Given concerns regarding students leaving school to purchase less healthy foods, we examined student behaviours and competitive food retail around schools in a large urban region of Southern Ontario. Methods: Using a geographic information system (GIS), we enumerated food outlets (convenience stores, fast-food restaurants, full-service restaurants) within 500, 1000 and 1500 m of all 389 regional schools spanning years of policy implementation. Consenting grade 6–10 students within 31 randomly selected schools completed a web-based 24-h diet recall (WEB-Q) and questionnaire. Results: Food outlet numbers increased over time (*p* < 0.01); post-policy, within 1000 m, they averaged 27.31 outlets, with a maximum of 65 fast-food restaurants around one school. Of WEB-Q respondents (*n* = 2075, mean age = 13.4 ± 1.6 years), those who ate lunch at a restaurant/take-out (*n* = 84, 4%) consumed significantly more energy (978 vs. 760 kcal), sodium (1556 vs. 1173 mg), and sugar (44.3 vs. 40.1 g). Of elementary and secondary school respondents, 22.1% and 52.4% reported ever eating at fast food outlets during school days. Conclusions: Students have easy access to food retail in school neighbourhoods. The higher energy, sodium and sugar of these options present a health risk.

## 1. Introduction

With nearly a third of 6- to 17-year old children and adolescents in Canada being overweight or obese [1], schools have an important role to play in supporting the health of children and adolescents. Approximately one third of dietary energy is consumed during the school day [2]. Moreover, the school environment can be influential on the development of health behaviours such as healthy eating, which can continue into adulthood [2,3,4]. In 2011, the province of Ontario’s Ministry of Education implemented the School Food and Beverage Policy (P/PM 150) in all publicly funded elementary and secondary schools, which set nutritional standards for all foods offered for sale in these environments (e.g., in cafeterias, tuck shops, vending machines, school programmes and events) [5]. Foods were categorized as *Sell Most*, *Sell Less* and *Not Permitted for Sale* according to their respective levels of health-promoting essential nutrients and nutrients of concern: fat, sugar and/or sodium. *Sell Most* choices were mandated to represent a minimum of 80% of foods offered for sale, *Sell Less*, no more than 20% and *Not Permitted for Sale* items, such as candies, soft drinks/soda and deep-fried foods, were banned.

The policy was mandated without consultation with students or those responsible for implementing it. School stakeholders have faced challenges establishing P/PM 150 and have voiced concerns that not all foods available for purchase are compliant [5,6,7,8]. Moreover, it was recognized that the policy of healthy food offered for sale at school needed to be supported by learning and social environments that engaged students around healthy eating [9]. Otherwise, students might opt for less healthy choices within lunches brought to school or purchased outside of school. Indeed, the influence of competitive food retail in school neighbourhoods on student eating behaviours is increasingly recognized [10,11,12,13,14]. For example, Laxer and Janssen [14] found that youth from schools with moderate or high density of fast-food restaurants were more likely to be excessive fast-food consumers than those from neighbourhoods without fast-food restaurants. Schools in lower-income areas have also been shown to have higher density of unhealthy food sources than high-income neighbourhoods [15,16,17]. With potentially less appealing food choices available at school, compounded with the fact that many students are permitted to leave school grounds during the school day, the policy impact of P/PM 150 could be weakened. 

The provincial implementation of P/PM 150 did not include a formal process evaluation to measure the delivery of the policy during its implementation. Public Health in a large urban region of Southern Ontario, however, took an active role in supporting schools over the early years (2010–2012) of establishing the policy. To gain a broad sense of what was working well and what further action may be required to support healthy eating in schools, they partnered with the University of Waterloo to examine the implementation within regional schools. This comprehensive process evaluation included surveillance of student eating behaviours; interviews and focus groups with school stakeholders; enumeration of food retail density around all regional schools; and investigation of the school food environment. Through surveying student food behaviours, it was possible to identify the impact of food purchase locations on students’ eating habits and nutritional intake. This has been a gap in many existing studies of student intake. Thus, the objective of this study was to examine eating behaviours from a cross-section of students from a large urban region of Southern Ontario in relation to where foods were procured during the school day and to examine the prevalence of competitive food retail surrounding all schools within the region over the period of implementation of the school food and beverage policy.

## 2. Materials and Methods

Data on student eating behaviours and food purchasing patterns were collected across early years of policy implementation (April 2012 to June 2013) from a random subset of elementary and secondary schools within each school board (Public and Catholic). School selection was based on geographic and economic distribution across the region with a desire to recruit a minimum of 2000 students. Hence, the sample was based on probability sampling of the total number of schools within each of three cities in the region, socioeconomic status (with equal numbers of school neighbourhoods below and above the regional median family income after tax from the most recent census data) and school level (elementary schools; secondary schools). A selection of 52 schools was identified, and the principals contacted from which 31 schools (60%) agreed to participate (19 elementary schools and 12 secondary schools). 

### 2.1. Data Collection

#### 2.1.1. Web-Based Eating Behaviour Questionnaire

Participants in grades 6 through 10, who provided written parental consent and personal assent, completed the validated University of Waterloo web-based eating behaviour questionnaire (WEB-Q) [18,19]. This tool included a 24-h food recall survey of about 900 common foods, food frequency questionnaire, and survey questions related to habitual diet–related behaviour—including frequency of purchase of foods from convenience store and fast food outlets. Energy drinks and shots were included, based on public health concern [20]. Data on Canada’s Food Guide food groups and nutrient intakes, as last specified in 2007 [21], were based on Canadian Nutrient File definitions and database at the time [22]. 

#### 2.1.2. Food Outlet Data

Food outlets in year spanning the initiation of the policy (2008, 2010, and 2012) were identified according to Food Inspection lists from Regional Public Health [23]. Collecting the data in multiple years allowed the assessment of changes in the food retail density over the span of policy implementation in relation to school neighbourhood mean family income. Food outlets were reclassified according the North American Industry Classification System (NAICS) definitions of convenience stores, limited service eating places (herein referred to as fast food restaurants), full-service restaurants and grocery stores/supermarkets [24]. 

Using the Geographic Information System (GIS) software, ArcGIS 10.0 (ESRI, 2012, Toronto, ON, Canada), schools (*n* = 380, 381 and 389 in 2008, 2010 and 2012, respectively) and food outlets were geocoded based on their street addresses. Three circular buffers (500, 1000 and 1500 m) [25] were created around each participating school. These demarcations were chosen to represent feasible walking distances for students, enabling to compare with Canadian studies using the 1 km buffer [11,12,13,14,26] and to bridge the 0.25 to 1 mile buffers (400, 800 and 1600 m) used in some US literature [27]. Although with slightly poorer goodness-of-fit [13], circular buffering was the most common approach at the time of our analysis and provides similar results to road network buffering. The numbers of each type of food outlets surrounding all regional schools within the buffering zones were calculated with the point-in-polygon tool in ArcGIS.

#### 2.1.3. Socio-Economic Data

To examine whether socioeconomic status was related to retail food density, data on median family incomes were extracted from the most current Statistics Canada’s census data for each school region’s dissemination area (2006 census in relation to 2008 and 2010 GIS data; 2011 census data for 2012) [28]. 

### 2.2. Statistical Analysis

Data from the WEB-Q were extracted and results separated by sex, age group and/or school level, where appropriate. Frequencies, percentages, averages and ranges were reported. Regression analysis was used to examine associations between mealtime location and food group and nutrient intakes. Using the GIS data, regression analysis was conducted to compare food outlet numbers with SES data.

### 2.3. Ethical Considerations

This study received full ethical clearance from the University of Waterloo Research Ethics Board (ORE# 16725), along with the research ethics committees of the regional public and Catholic school boards. In order to protect the identity of invited and participating schools, all school names were removed from documentation. Participating students used unique, one-time login and password codes; survey responses could not be traced back to individual students.

## 3. Results

### 3.1. WEB-Q

From a total of 2102 students who consented to participate in the WEB-Q, 2075 students (elementary = 1188 and secondary = 887; mean age 13.4 ± 1.6 years) were included in the analysis after excluding participants who did not report age, sex or grade. Demographic traits for the participants are given in Table 1. 

**Figure 1:**
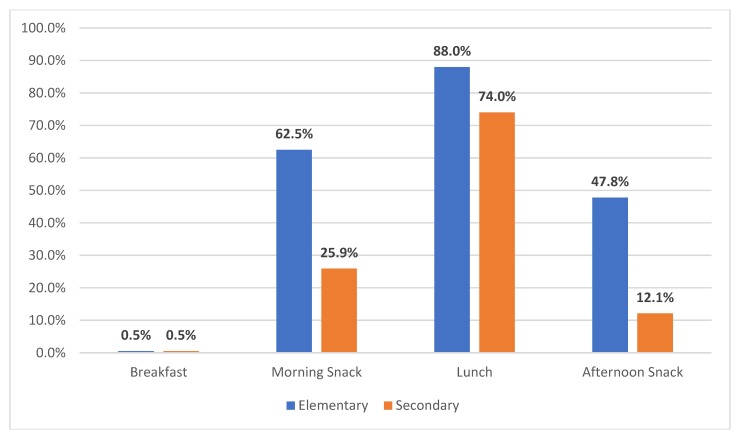
Proportion of elementary and secondary students eating snacks/meals at school on the day of the 24-h diet recall.

On the day of the 24-h recall, most students ate lunch at school (elementary = 88.0% and secondary 74.0%) (Figure 1). 

Lunch intakes of students who ate at a restaurant/take-out on the day of the recall (*n* = 84, 4%) were significantly higher than those of other students (*n* = 1991) for energy (978 versus 760 kcal), sodium (1556 versus 1173 mg) and total sugars (44.3 versus 40.1 g) (based on 95% confidence intervals, data adjusted for age and gender). Many student bring food from home; habitually, 39.1% of secondary school students ate at the school cafeteria at least once a week, while 41.9% of the full sample rarely or never ate at the cafeteria (Table 2). About 35.6% of students ate meals prepared from fast-food restaurants at least once per week, although the specific meal occasion was not specified. While some students ate from vending machines (13.4%, *n* = 263) and tuck shop/snack bars (16.9%, *n* = 334) at least once a week, most did so rarely or never (66.1%, *n* = 1301 and 48.0%, *n* = 948, respectively) (Table 2). 

**Figure 2:**
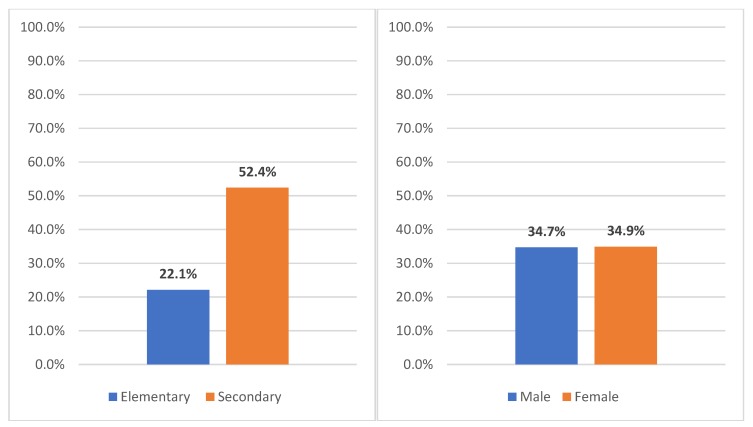
Proportion of elementary and secondary students ever buying lunch at a fast food restaurant/take-out during the school day.

About half of the secondary students (52.4%, *n* = 442) and a fifth of elementary students (22.1%, *n* = 257) responded ’yes’ to the question, “Do you ever buy food during the school day at a restaurant or take-out?” (Figure 2). While we didn’t specify a time frame, the use of present tense suggests respondents wouldn’t have referred to the distant past. 

Participants were asked about the habitual consumption of a list of foods and beverages, including a list of “other” foods that were high in energy density and low in nutritional value. These type of foods and beverages were consumed at least twice a week by students, including regular soft drinks (39.5%, *n* = 785), candy or chocolate bars (44.2%, *n* = 874), and salty snack foods such as chips (55.8%, *n* = 1117) (Table 3). 

### 3.2. Retail Food Density versus Socio-Economic Status

In 2012, the average number of fast-food, full-service restaurant and convenience store food outlets was high (a mean of 12.2, 6.4 and 7.0 at 1000 m, respectively) with a maximum of 36 and 65 fast food outlets, within 500 and 1000 m, respectively (Table 4). There was a significant increase of total food retail density over the years 2008, 2010 and 2012 at both 1000 m (25.4, 26.3 and 27.3 mean outlets per school, respectively) and 1500 m buffers (56.2, 58.8 and 61.8 mean outlets per school, respectively) (*p* < 0.01 each). There was a negative association between the average number of food retail outlets (inclusive of convenience stores, fast food restaurants, full-service restaurants and supermarkets) and mean family income at all buffer zones for each of 2008, 2010, and 2012 (r = −0.16 to −0.40, *p* < 0.01 each).

## 4. Discussion

This current study demonstrated that students often opt to purchase food in school neighbourhoods, of which there are many food retail choices. About a quarter of secondary students did not eat their lunch at school on the day of the 24-h recall, a habit that was more common than in elementary students. Ontario principals have voiced concern that the requirement for healthier food options at school is both reducing cafeteria profits and driving students to purchase foods outside of school [7]. Seliske et al. [13] had also observed that about a third of grade 9–10 students usually did not eat lunch at school as secondary students have more freedom to leave school grounds during lunch time. Staying at school to eat lunch prepared from home or school cafeteria is more likely to provide a healthier meal [30], and thus, there have been intervention programs to try to encourage students to stay at school for lunch [31,32]. Over a third of students in our study ate meals prepared from fast-food restaurants at least once per week. This figure is much higher than the rate reported by Canadian youths (aged 11–15) studied by Laxer and Janssen [14], but lower than what was observed by Lillico et al. [33] among students in grade 5–12, possibly due to the difference between our regional sample and the varied cross-Canada contexts of the other studies. Students who ate fast food for lunch in our study consumed significantly more calories, sodium and sugar compared to other students. This finding is not surprising, as the consumption of fast food has been linked to poorer dietary quality [34], and fast food proximity to schools has been associated with overweight in adolescents [35,36], though this could reflect other factors, such as home neighbourhood location [37]. 

Aside from the external environment, students may also have access to other sources of unhealthy food within schools. Prior to provincial school food and beverage policies, vending machines could provide energy-dense foods that students may elect to consume and defer nutrient rich fruits and vegetables [38,39,40,41]. Even after P/PM 150, full adherence to policy within vending offerings was uncommon [8,9]. The majority (about two-thirds) of students in our study stated that they rarely or never ate food from a vending machine, although about 13 percent did so at least once a week. Further work is required to ensure healthy eating habits among students by improving the nutritional quality of products available for sale in these machines both within schools and in other settings, like recreation facilities, where children spend time [42].

We found a significant increase in retail food density at the 1000 and 1500 m buffering zones across the span of introducing the policy (2008 to 2012). While this change may be independent of the policy, it nevertheless reflects the food environment to which students are exposed ear their schools. The average number of food retail outlets was also significantly higher around schools in lower-income areas. This finding is consistent with the literature [14,15,16,17], and the availability of these food environments has been shown to be strongly related to students’ eating behaviours during the school day [13]. The connection between unhealthy food outlets in low-income neighbourhoods and increased likeliness of children being overweight or obese has also been observed in Toronto, Ontario [43]. Furthermore, children attending schools in low-income areas have been shown to have greater objectively measured accessibility to fast-food outlets as they move throughout the day. In other words, even when taking account of mobility patterns of these children during the day, those from low-income areas have easier access to unhealthy food outlets [15]. Thus, specific population groups may require additional attention in regard to the implementation of school policy to support its role in promoting healthy eating. 

Our data showing frequent student consumption of “other” foods, like soda, French fries, salty snacks and candy, underlies the importance of policies like P/PM 150 that limit access to such energy-dense, nutrient-poor foods, at least on school property. This also underlies the importance of monitoring and evaluating school policy, since concerns with adherence, at least within vending machine offerings, have been noted through our own research [9] and that of others [8]. Frequent consumption of energy-dense, nutrient-poor foods is consistent with other studies of Canadian youth [2,33] and, perhaps, not surprising given their ubiquitous availability arounds schools and neighbourhoods, certainly in urban environments [12,13,14,15,16,17,36]. This suggests that if the intent of policies like Ontario’s School Food and Beverage Policy is to enhance student diets and health, schools need to adopt more comprehensive approaches encompassing policy options beyond foods offered for sale and extending to teaching and learning, social and physical environments and partnerships with family and community [44]. At present, there are no bylaws to restrict food retail around schools in Ontario. While such changes are complex [44], zoning bylaws to ban fast-food drive-through services have been implemented in some jurisdictions across Canada, suggesting the potential for regulation of school neighbourhood retail environments to support healthier eating. 

### Strengths and Limitations

A key strength of this study included the use of a nutrition assessment tool (WEB-Q) to examine the eating behaviour and nutrient intake of students in relation to competitive food retail. Through this tool, we examined both the frequency and specific type of foods consumed by elementary and secondary school students. Nevertheless, this was a cross-sectional study and may not be an accurate representation of students’ habitual consumption or food outlet choices. Self-response may have introduced bias. For example, only two-thirds of respondents provided both height and weight data, and while the prevalence of overweight for the sample compares with national data for self-reported measures, the prevalence of obesity at 4.5% was less than half that expected [1]. Another limitation was the estimation of socio-economic status based on the mean family income from school region census data and not specifically families of students attending each school. Observing food retail density spanning the time of policy implementation gave some appreciation of the shifting nature of food retail. While it would have been desirable to repeat the analyses, funding was not available. Nevertheless, given the absence of municipal bylaws affecting food retail around schools, the situation is unlikely to have improved. Our analysis over the years of implementation of P/PM 150 [9,43] and 2014 data on vending compliance in Ontario and Alberta [9] provide the most current appraisal of this policy. 

## 5. Conclusions

This study adds to the current evidence that many students, particularly those attending secondary school, leave school grounds during the school day to purchase food. Students who consumed fast food or take-out for lunch showed a higher intake of calories, fats and sugar than other students. The growing number of food retail outlets around schools over the time of implementation of school policy is of concern, especially for this urban region of Southern Ontario where the density of fast food outlets was already high, particularly around schools in low-income neighbourhoods. The policy impact of P/PM 150 is limited by the relatively small numbers of students who select foods offered for sale at school and by the lack of attention to broader social and environmental factors that influence food choices of students.

## Figures and Tables

**Table 1:** Demographic characteristics of students included in the evaluation of Ontario’s School Food and Beverage Policy (*n* = 2075 students).

Characteristic	Number of Students (%)
School Type	
Elementary	1188 (57.3%)
Secondary	887 (42.8%)
Sex	
Male	943 (45.4%)
Female	1132 (54.6%)
Grade	
6	408 (19.7%)
7	406 (19.6%)
8	374 (18.0%)
9	495 (23.9%)
10	392 (18.9%)
Body Mass Index Category *	
Underweight	37 (2.6%)
Healthy Weight	1084 (75.5%)
Overweight	250 (17.4%)
Obese	65 (4.5%)

* Based on self-report and WHO standards [29]; 1436 participants (68.2%) reported both height and weight from which BMI was calculated.

**Table 2:** Frequency of elementary and secondary students eating meals or snacks prepared away from home.

Eating Meals or Snacks Prepared from…	*n* of Students Eating at Reported Frequency (%)				
	2–6 Times a Week	Once a Week	Once a Month	Rarely or Never	Total
School Cafeteria	295 (14.9%)	491 (24.8%)	364 (18.4%)	828 (41.9%)	1978
Vending Machines	89 (4.5%)	174 (8.9%)	403 (20.5%)	1301 (66.1%)	1967
Tuck Shop/Snack Bar	121 (6.1%)	213 (10.8%)	692 (35.1%)	948 (48.0%)	1974
Convenience Stores	185 (9.4%)	309 (15.7%)	628 (31.9%)	850 (43.1%)	1972
Friend/Relative’s Home	302 (15.2%)	534 (26.9%)	748 (37.7%)	401 (20.2%)	1985
Fast Food Restaurants	183 (9.2%)	522 (26.4%)	901 (45.5%)	375 (18.9%)	1981
Other Restaurants	87 (4.5%)	267 (13.7%)	1029 (52.7%)	568 (29.1%)	1951

**Table 3:** Habitual consumption of “other” foods by elementary and secondary students based on the FFQ.

Type of Food	*n* of Students Eating at Reported Frequency (%)					
	At Least Once a Week		2–4 Times a Month		Rarely/Never	
	Elementary	High School	Elementary	High School	Elementary	High School
Pop/soda (non-diet)	424 (36.8%)	361 (43.3%)	341 (29.6%)	230 (27.6%)	386 (33.5%)	243 (29.1%)
French fries or other fried potatoes	330 (28.6%)	327 (39.1%)	549 (47.7%)	374 (44.7%)	273 (23.7%)	136 (16.2%)
Pizza	299 (25.8%)	237 (28.3%)	698 (60.3%)	489 (58.4%)	160 (13.8%)	112 (13.4%)
Candy or chocolate bars	499 (43.4%)	378 (45.3%)	380 (33.0%)	294 (35.3%)	272 (23.6%)	162 (19.4%)
Salty snacks (e.g., chips)	632 (54.3%)	485 (57.5%)	305 (26.2%)	220 (26.1%)	226 (19.4%)	138 (16.4%)
Energy shots (e.g., 5-h energy^®^)	49 (4.3%)	20 (2.4%)	35 (3.0%)	30 (3.6%)	1066 (92.7%)	789 (94.0%)
Coffee-based energy drinks (e.g., Rockstar roasted^®^)	92 (8.0%)	125 (15.0%)	143 (12.4%)	134 (16.0%)	919 (79.6%)	576 (69.0%)
Energy drinks (e.g., NOS^®^, Red Bull^®^, Monster^®^)	61 (5.3%)	41 (4.9%)	94 (8.1%)	96 (11.5%)	1002 (86.6%)	700 (83.6%)

**Table 4:** Food retail outlets within 500, 1000 and 1500 m of all regional schools.

Year		2008	2010	2012
Food Outlets by Buffer Zone		Mean Number of Outlets (Range)		
500 m	Convenience	1.39 (0–25)	1.31 (0–21)	1.32 (0–20)
	Fast Food	2.07 (0–30)	2.25 (0–37)	2.30 (0–36)
	Full Serve Rest.	1.13 (0–26)	1.09 (0–22)	1.14 (0–23)
	Supermarket	0.36 (0–5)	0.35 (0–6)	0.34 (0–6)
	TOTAL	4.95 (0–86)	5.00 (0–86)	5.10 (0–85)
1000 m	Convenience	6.93 (0–35)	6.81 (0–30)	7.03 (0–32)
	Fast Food	10.90 (0–51)	11.66 (0–61)	12.17 (0–65)
	Full Serve Rest.	5.77 (0–39)	6.13 (0–40)	6.38 (0–42)
	Supermarket	1.76 (0–10)	1.75 (0–10)	1.73 (0–11)
	TOTAL	25.35 (1–127)	26.34 (1–127)	27.31 (1–233)
1500 m	Convenience	14.90 (0–56)	14.75 (0–52)	15.25 (0–53)
	Fast Food	23.76 (0–82)	25.65 (0–93)	27.28 (0–105)
	Full Serve Rest.	13.71 (0–68)	14.53 (0–65)	15.30 (0–68)
	Supermarket	3.82 (0–16)	3.85 (0–17)	3.97 (0–16)
	TOTAL	56.19 (1–210)	58.78 (1–216)	61.8 (1–233)

Data are for all schools (*n* = 380 in 2008, *n* = 381 in 2010, *n* = 389 in 2012). Note that the numbers represent that average of the results by school by category. Hence, individual store count and score totals may differ. Total food density around schools increased over time at 1000 and 1500 m buffer zones (*p* < 0.01).
